# Effectiveness of rapid rule-out strategy for acute coronary syndrome in the emergency department: a real-world retrospective study in Asia

**DOI:** 10.3389/fcvm.2025.1697660

**Published:** 2025-12-09

**Authors:** Peng Zhang, Lianlian Cao, Mei Ren

**Affiliations:** Department of Emergency, Dongying People’s Hospital, Dongying, Shandong Province, China

**Keywords:** cardiac troponin T protein, emergency medicine, myocardial infarction, acute coronary syndrome, emergency

## Abstract

**Background:**

The European Society of Cardiology (ESC) 0/1 h high-sensitivity cardiac troponin T (hs-cTnT) algorithm is endorsed for rapid triage of patients with suspected acute coronary syndrome (ACS). However, its real-world performance, particularly in Asian populations and among patients with comorbidities, remains uncertain.

**Methods:**

We conducted a retrospective observational study of consecutive patients presenting with suspected ACS to the emergency department of a tertiary hospital in China between May 2023 and May 2025. Patients were classified by the ESC 2020 0/1 h hs-cTnT algorithm into rule-out, observe, or rule-in groups. The primary outcome was the diagnostic performance of the rule-in arm, assessed by specificity and positive predictive value (PPV) for myocardial infarction (MI) during admission. Disposition decisions and length of stay (LOS) were also evaluated. A sensitivity analysis compared the ESC 2015 and 2020 thresholds.

**Results:**

A total of 508 patients were included (median age 62 years, 39% women). According to the 2020 ESC algorithm, 203 patients (40%) were classified as rule-out, 254 (50%) as observe, and 51 (10%) as rule-in. In the rule-in group, 42 patients had a final diagnosis of MI, yielding a PPV of 82%. None of the rule-out patients were diagnosed with MI, although 20% were admitted despite meeting rule-out criteria. The observe group was clinically heterogeneous, with 17% diagnosed with MI and the longest ED LOS (median 7.4 h). Patients with known coronary artery disease were more often classified as rule-in or observe, whereas younger patients were predominantly rule-out. In the sensitivity analysis, applying 2015 thresholds reduced the rule-in proportion and increased the observe group, without improving discrimination for MI.

**Conclusions:**

The ESC 0/1 h hs-cTnT algorithm demonstrated good diagnostic concordance in the rule-in group and high safety in the rule-out group, but nearly half of the patients remained in the observe category, limiting operational efficiency. The 2020 threshold adjustments reclassified more patients as rule-in or rule-out but did not enhance diagnostic yield. These findings suggest that while the algorithm can support early decision-making, its thresholds may require recalibration to local populations and clinical practice settings.

## Introduction

Acute myocardial infarction (AMI) remains a leading cause of emergency hospitalisation and mortality worldwide (1). Rapid and accurate recognition of MI in patients presenting to the emergency department (ED) is critical to guide early reperfusion therapy and evidence-based treatment (2). At the same time, most patients who present with chest pain or related symptoms are not ultimately diagnosed with MI (1). Routine admission of these low-risk patients contributes to ED crowding, unnecessary testing, and increased healthcare costs. Efficient triage strategies are therefore essential, enabling safe early discharge of patients without MI while ensuring prompt identification and management of those with confirmed disease.

The development of high-sensitivity cardiac troponin (hs-cTn) assays has improved the diagnostic approach to suspected acute coronary syndrome (ACS) (3). The European Society of Cardiology (ESC) introduced the hs-cTn 0/1 h algorithm (4), which uses troponin values at presentation and 1 h to classify patients into rule-out, observe, or rule-in categories. This approach has been incorporated into both ESC and American Heart Association/American College of Cardiology guidelines as a recommended tool for the evaluation of non-ST-segment elevation MI (5, 6).

Large validation studies, conducted predominantly in Europe and Australasia, have shown that the ESC 0/1 h algorithm can rapidly classify a substantial proportion of patients with high diagnostic safety, particularly in the rule-out arm (7–9). However, performance has been less consistent in other settings. In North American cohorts, for example, the algorithm has been associated with higher rates of missed diagnoses compared with European studies (10). These discrepancies may reflect differences in baseline risk profiles, prevalence of coronary artery disease (CAD), and healthcare pathways (11). Importantly, CAD itself remains a key determinant of risk in patients presenting with suspected ACS, yet the ESC 0/1 h algorithm does not incorporate clinical variables such as CAD history into its classification (12). Evidence from observational studies suggests that diagnostic accuracy, particularly in the rule-out group, may be reduced in patients with established CAD (13).

Although the ESC 0/1 h algorithm has been widely introduced, much of the evidence comes from prospective trial cohorts with strict protocol adherence. Real-world data on its performance in routine care, where practice is shaped by workload and clinical judgment, are sparse. Little is known about how patients classified as rule-out are subsequently managed and whether the algorithm meaningfully reduces emergency department length of stay (LOS).

This study aimed to evaluate the potential impact of the ESC 0/1 h hs-cTnT algorithm in a real-world ED visiting population. The primary objective was to determine the diagnostic concordance of the rule-in arm by assessing its specificity and positive predictive value (PPV) for MI during admission. Secondary objectives were to examine the management of patients triaged to the rule-out arm and to explore its potential impact on emergency department efficiency through analysis of LOS.

## Methods

### Study design and setting

This was a retrospective, single-centre observational study conducted in the emergency department of Dongying People's Hospital in China. The study period is from 03 May 2023 to 16 May 2025, after the introduction of ESC 0/1 hs-cTn testing into routine clinical practice at our hospital. Patients presenting with suspected ACS underwent baseline and 1 h hs-cTnT testing, with classification according to the ESC 0/1 h algorithm to guide initial triage. When the diagnosis remained uncertain, or in patients presenting very early after chest pain onset, a 3 h sample was performed in line with guideline recommendations. Final admission and discharge decisions were determined by the treating clinicians, who also considered ECG findings, comorbidities, and overall clinical assessment. Our study follows the STROBE guidelines.

### Study population

We included consecutive patients aged 18 years or older who presented to the emergency department with symptoms concerning for ACS, including chest pain, dyspnoea, or angina-equivalent presentations, and who underwent serial hs-cTn testing within 1 h of arrival. Patients receiving chronic dialysis, pregnant and breastfeeding women, and patients with missing baseline and 1 h serial hs-cTn measurements were also excluded.

### Data collection

Clinical data were retrospectively extracted from the hospital's electronic records, and hs-cTn data were collected from the stored lab records. Demographic variables included age, sex, and smoking status (current, former, or never). Clinical history was obtained for hypertension, diabetes mellitus, hyperlipidaemia, coronary artery disease (including prior myocardial infarction, percutaneous coronary intervention, or coronary artery bypass grafting), peripheral vascular disease, congestive heart failure, abdominal aortic aneurysm, atrial fibrillation, chronic kidney disease, chronic lung disease, and prior stroke. Presenting characteristics included the time from chest pain onset or peak to emergency department presentation and the time from symptom onset to the first blood draw. Electrocardiograms (ECG) were reviewed and categorised as with and without ischaemic changes. ECGs were categorised as ischaemic if they demonstrated any of the following findings suggestive of acute myocardial ischaemia: new or dynamic ST-segment depression ≥0.5 mm, ST-segment elevation ≥1 mm in two contiguous leads (excluding clear non-ischaemic causes), or T-wave inversion ≥1 mm in two contiguous leads. ECGs without these changes, including those showing non-specific ST–T abnormalities, left bundle branch block without new changes, paced rhythms, or normal findings, were classified as non-ischaemic. Baseline and 1 h hs-cTn concentrations were reported, and 3 h results were available.

### ESC 0/1 h algorithm

hs-cTnT was quantified using the Elecsys Troponin T Gen 5 STAT assay on the Cobas e 601 analyser (Roche Diagnostics, Basel, Switzerland). The analytical range of the assay is 3–10,000 ng/L, with a limit of detection of 5 ng/L, a limit of quantification of 6 ng/L, and a 99th percentile upper reference limit of 14 ng/L (14).

In the main analysis, we used the 2020 ESC thresholds (4). Patients were ruled out if the baseline hs-cTnT was <4 ng/L, chest pain onset was >3 h before presentation, or the baseline hs-cTnT was <12 ng/L with an absolute 0–1 h change <2 ng/L. Patients were ruled in if the baseline hs-cTnT was ≥64 ng/L or if the 0–1 h absolute change was ≥6 ng/L. All others were assigned to the observe zone.

In the sensitivity analysis, we applied the 2015 ESC 0/1 h thresholds (15). Rule-out was defined as a baseline hs-cTnT <2 ng/L with chest pain onset >3 h or a baseline hs-cTnT <12 ng/L with an absolute 0–1 h change <2 ng/L. Rule-in was defined as a baseline hs-cTnT ≥52 ng/L or an absolute 0–1 h change ≥6 ng/L. All other patients were classified into the observation group. As 99th percentile URL values were not available in our dataset, the 0/3 h pathway recommended in the 2015 ESC guidelines could not be evaluated.

### Outcomes

The primary outcome was the diagnostic performance of the rule-in arm of the 2020 ESC 0/1 h algorithm, expressed as its specificity and positive predictive value for MI diagnosis during the ED admission. All final diagnoses of MI were confirmed by the clinician team, with adjudication based on chart review, serial investigations, and consensus of treating cardiologists. The secondary outcomes were the management of patients triaged to the rule-out arm and the ED length of stay. The management of rule-out patients was assessed by determining the proportion discharged directly from the ED compared with those admitted despite meeting rule-out criteria. ED LOS was defined as the interval from ED registration to the admission or discharge decision and was examined across all triage groups.

### Statistical analysis

Continuous variables were summarised as mean (standard deviation) or median (interquartile range), and categorical variables were expressed as counts and percentages. Group differences in baseline characteristics were assessed using the Mann–Whitney *U* test for continuous variables and the *χ*^2^ test or Fisher's exact test for categorical variables. The diagnostic performance of the ESC 0/1 h algorithm was assessed by calculating specificity and positive predictive value, with exact 95% confidence intervals (95% CI). For the secondary outcome, ED LOS was compared across rule-out, observe, and rule-in groups and presented graphically in the bar plot.

Subgroup analyses were conducted in patients with and without known CAD and in patients over 55 years and younger than 55 years old. In sensitivity analyses, the 2015 ESC thresholds were applied to assess whether the updated 2020 criteria, used in the primary analysis as the current clinical standard, provided additional diagnostic benefit or improved rule-out precision in our population. All tests were two-tailed, and *p* < 0.05 was considered statistically significant without adjustments for multiple testing [R version 4.4.1 (R Foundation for Statistical Computing, Vienna, Austria)].

## Results

**Figure 1:**
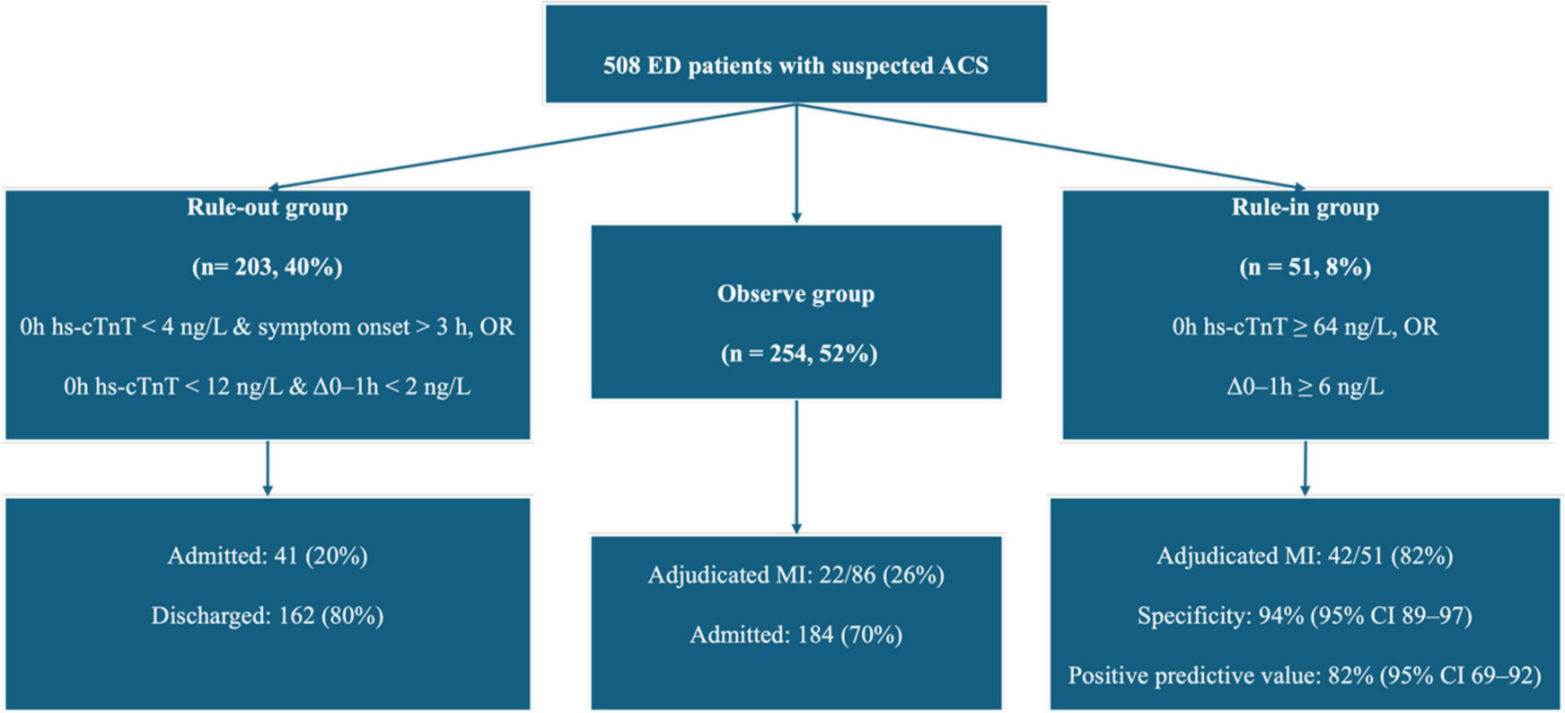
Patient classification by the ESC 0/1 h hs-cTnT algorithm.

The study included 508 patients with suspected ACS, of whom 196 (39%) were women and 312 (61%) men, with a median age of 62 years (IQR: 54–70). According to the 2020 ESC 0/1 h algorithm, 203 patients (40%) were classified as rule-out, 254 (50%) as observe, and 51 (10%) as rule-in (Figure 1). Patients in the rule-in group were older and more likely to have a history of CAD and diabetes compared with those in the rule-out and observe groups. Ischaemic ECG changes were present in 78% of rule-in patients, 39% of those in the observe group, and 6% of rule-out patients, with a significant difference in ECG patterns across groups (*p* < 0.001). Baseline hs-cTnT concentrations were highest in the rule-in group (median 22.1 ng/L, IQR: 13.3–32.5) and lowest in the rule-out group (7.9 ng/L, IQR: 6.0–9.7), with significant variation across groups (*p* < 0.001). Other traditional cardiovascular risk factors, including hypertension and hyperlipidaemia, were common across groups but did not differ significantly (Table 1).

**Table 1:** Baseline table.

Variable	Overall (*n* = 508)	Rule-out (*n* = 203)	Observe (*n* = 254)	Rule-in (*n* = 51)	*p*-value
Age, median (IQR)	62.0 (54.0–70.0)	60.0 (50.0–67.0)	63.5 (55.0–71.0)	68.0 (59.0–75.0)	<0.001*
Sex					0.30
Female, *n* (%)	196 (39%)	86 (42%)	93 (37%)	17 (33%)	
Male, *n* (%)	312 (61%)	117 (58%)	161 (63%)	34 (67%)	
Smoking					>0.90
Current smoking, *n* (%)	154 (30%)	61 (30%)	75 (30%)	18 (35%)	
Former smoking, *n* (%)	123 (24%)	49 (24%)	64 (25%)	10 (20%)	
Never smoked, *n* (%)	231 (45%)	93 (46%)	115 (45%)	23 (45%)	
Comorbidities					
Hypertension, *n* (%)	222 (44%)	86 (42%)	116 (46%)	20 (39%)	0.60
Diabetes mellitus, *n* (%)	117 (23%)	20 (10%)	80 (31%)	17 (33%)	<0.001*
Coronary artery disease, *n* (%)	115 (23%)	6 (3%)	76 (30%)	33 (65%)	<0.001*
Hyperlipidaemia, *n* (%)	163 (32%)	73 (36%)	75 (30%)	15 (29%)	0.30
Congestive heart failure, *n* (%)	69 (14%)	29 (14%)	36 (14%)	4 (8%)	0.50
Atrial fibrillation, *n* (%)	37 (7%)	11 (5%)	22 (9%)	4 (8%)	0.40
Chronic kidney disease, *n* (%)	52 (10%)	15 (7%)	34 (13%)	3 (6%)	0.061
Peripheral vascular disease, *n* (%)	28 (6%)	17 (8%)	8 (3%)	3 (6%)	0.046*
Prior stroke, *n* (%)	40 (8%)	10 (5%)	23 (9%)	7 (14%)	0.064
Chronic lung disease, *n* (%)	58 (11%)	23 (11%)	27 (11%)	8 (16%)	0.60
Abdominal aortic aneurysm, *n* (%)	16 (3%)	6 (3%)	8 (3%)	2 (4%)	0.90
eGFR, mL/min/1.73m², median (IQR)	75.0 (61.0–93.0)	75.0 (60.0–90.0)	78.0 (61.0–94.0)	80.0 (68.0–93.0)	0.30
ECG					<0.001*
Ischaemic, *n* (%)	152 (30%)	13 (6%)	99 (39%)	40 (78%)	
Non-ischaemic, *n* (%)	356 (70%)	190 (94%)	155 (61%)	11 (22%)	
Baseline hs-cTnT, ng/L, median (IQR)	12.4 (8.3–17.4)	7.9 (6.0–9.7)	15.7 (13.2–20.0)	22.1 (13.3–32.5)	<0.001*
Onset to ED arrival, h, median (IQR)	3.1 (2.2–4.1)	3.1 (2.2–4.1)	3.1 (2.2–4.2)	3.1 (2.1–4.0)	>0.90
Onset to first blood draw, h, median (IQR)	3.8 (2.9–4.8)	3.8 (2.8–4.9)	3.7 (2.8–4.8)	3.7 (3.0–4.7)	>0.90

IQR, interquartile range; eGFR, estimated glomerular filtration rate; hs-cTnT, high-sensitivity cardiac troponin T; ED, emergency department.

*p*-value: overall group differences among rule-out, observe, and rule-in groups.

* *p* < 0.05.

Of the 51 patients classified as rule-in by the algorithm, 42 had a final adjudicated diagnosis of MI, corresponding to a positive predictive value of 82%. The corresponding specificity for MI during admission was 94% (95% CI: 89–97). Among the 203 patients classified as rule-out, 162 (80%) were discharged from the ED, 41 (20%) were admitted, and none were subsequently diagnosed with MI. Of the 254 patients in the observe group, 76 (30%) were discharged, 178 (70%) were admitted, and 42 (17%) had a final adjudicated diagnosis of MI. All 51 patients in the rule-in group were admitted, of whom 42 (82%) were confirmed to have MI, while 9 (18%) did not (Figure 1).

**Figure 2:**
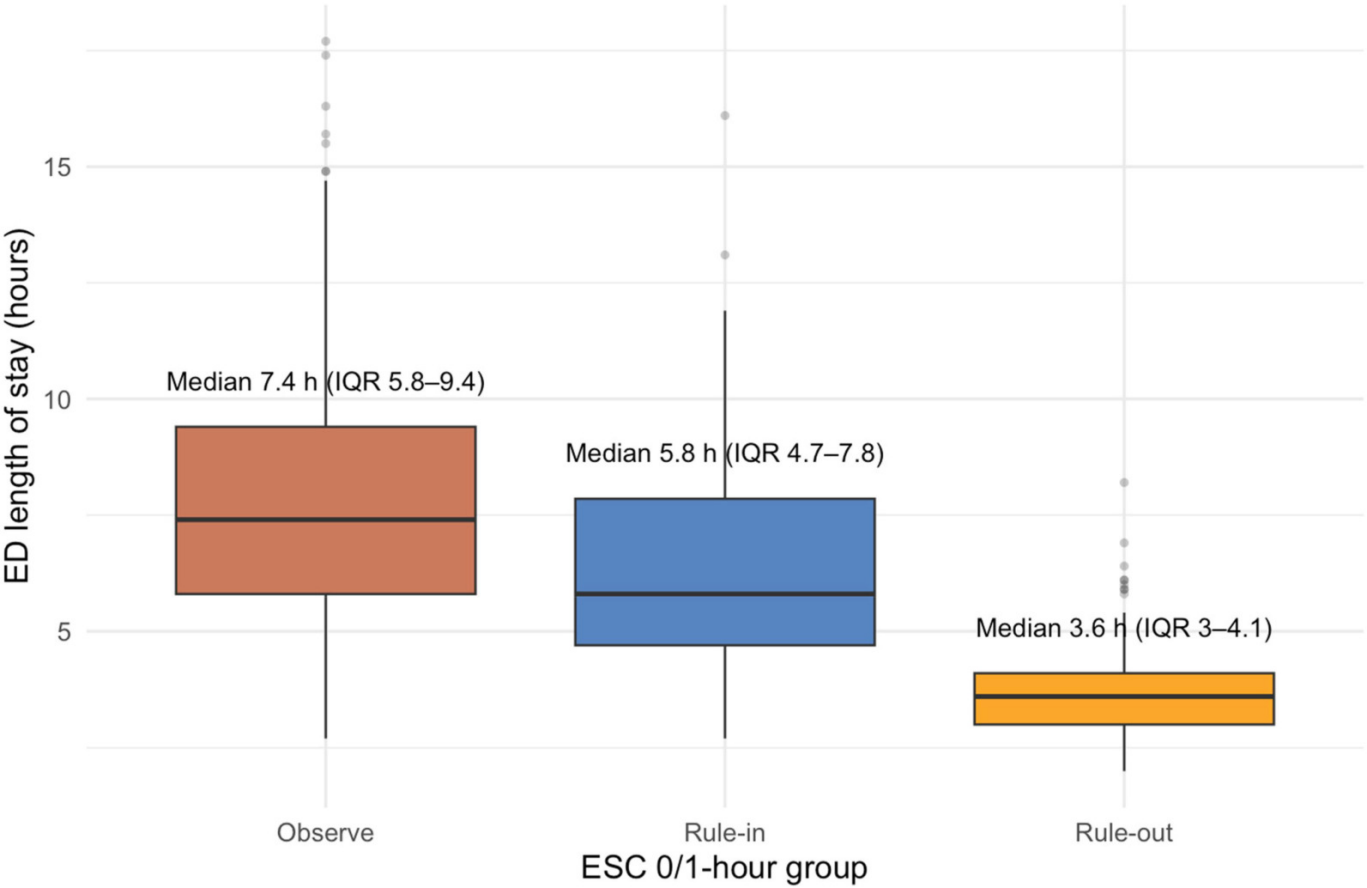
ED length of stay by ESC 0/1 h algorithm group.

Median length of stay differed across groups. Patients in the rule-out group had the shortest stay in the ED (median 3.6 h, IQR: 2.8–4.7), followed by the rule-in group (5.8 h, IQR: 4.1–7.3). The observe group experienced the longest stays (7.4 h, IQR: 5.6–9.9) (Figure 2).

Among the patients with coronary artery disease, 29% were classified as rule-in, 66% as observe, and only 5% as rule-out (Table 2). In contrast, among those without coronary disease, half were classified as rule-out, 45% as observe, and 5% as rule-in. Final adjudication of MI showed that 79% of rule-in patients with CAD and 75% without CAD had a confirmed diagnosis. The median overall ED LOS was longer in patients with CAD compared with those without [6.5 h (IQR: 4.5–8.3) vs. 5.2 h (IQR: 3.9–7.1)].

**Table 2:** Distribution of patients across ESC 2020 0/1 h algorithm groups by subgroups.

Subgroup	Rule-out, *n* (%)	Observe, *n* (%)	Rule-in, *n* (%)	Total (*n*)
Overall	203 (40%)	254 (50%)	51 (10%)	508
CAD present	6 (5.2%)	76 (66.1%)	33 (28.7%)	115
No CAD	197 (50.1%)	178 (45.3%)	18 (4.6%)	393
Age <55 years	76 (54.7%)	57 (41.0%)	6 (4.3%)	139
Age ≥55 years	127 (34.4%)	197 (53.4%)	45 (12.2%)	369

In patients younger than 55 years, 55% were classified as rule-out and 4% as rule-in, whereas in those aged 55 years or older, 34% were in the rule-out group, 53% in the observe group, and 12% in the rule-in group (Table 2). Final MI was found in 83% of younger and 82% of older patients assigned to the rule-in group. Median ED LOS was shorter among younger patients [5.0 h (IQR: 3.7–7.0)] compared with that of patients aged ≥55 years [6.1 h (IQR: 4.5–8.0)].

**Figure 3:**
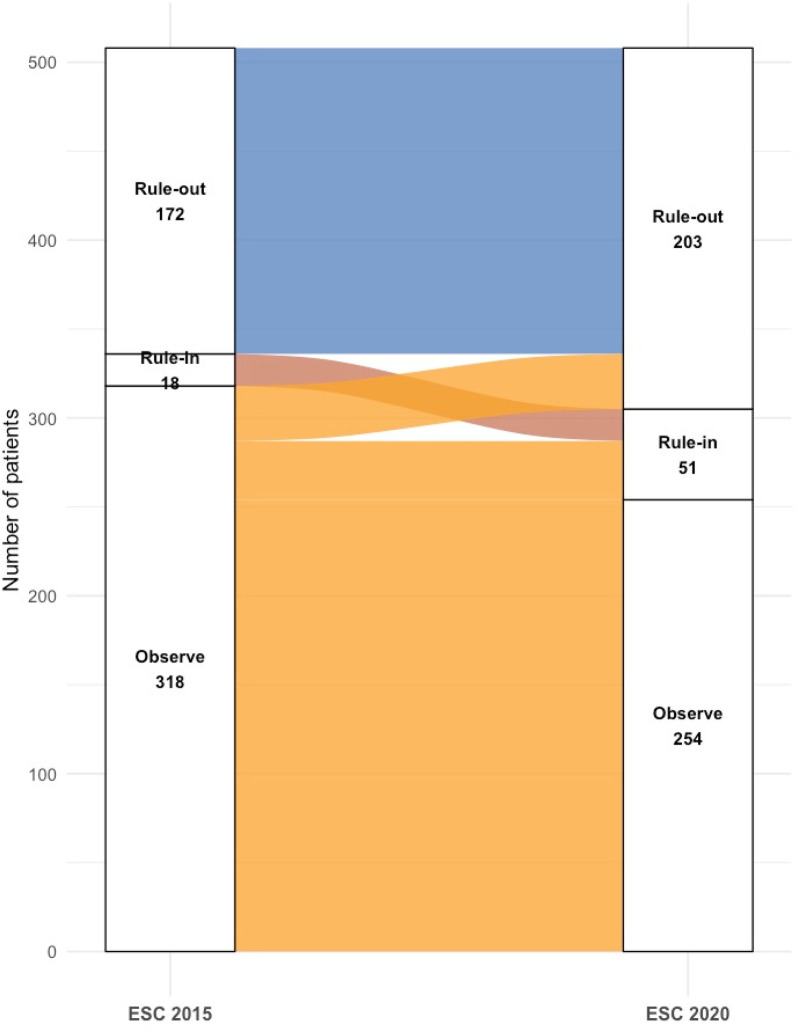
Reclassification of patients by ESC 2015 vs. 2020 0/1 h algorithms.

When the 2015 thresholds were applied to the same cohort (Figure 3), fewer patients were assigned to definitive categories: only 172 (34%) as rule-out and 18 (3%) as rule-in, with the majority (318, 63%) placed in the observation group. Relative to the 2020 classification, 31 patients moved from rule-out to observe and 33 from rule-in to observe. Under the 2015 criteria, the rule-in group demonstrated a specificity of 98%, compared with 93.8% using the 2020 thresholds.

## Discussion

The primary finding of this study is that the ESC 0/1 h hs-cTnT algorithm classified 40% of patients as rule-out, 50% as observe, and 10% as rule-in. In the rule-in arm, 82% were confirmed to have myocardial infarction, demonstrating good diagnostic concordance but leaving nearly one in five patients misclassified. In the rule-out group, 80% of patients were discharged directly from the ED, while 20% were admitted despite meeting rule-out criteria, reflecting the influence of comorbidities and clinical caution. Patients in the observe zone accounted for half of the cohort and had the longest median length of stay, underscoring the limited ability of the algorithm to provide definitive triage in this group.

The ESC 0/1 h algorithm performed broadly in line with prior reports, but with slightly higher PPV and specificity, showing several clinically important caveats (16, 17). In the rule-in arm, most patients were correctly identified as having myocardial infarction, yet nearly one in five were false positives. However, in practice, this means that nearly one-fifth of patients classified as confirmed ultimately do not develop an AMI. For these patients, hospital admission often leads to unnecessary additional testing and treatment, increasing patient risk and straining healthcare resources. This limitation reflects a fundamental characteristic of high-sensitivity troponin assays: While they are highly sensitive for detecting myocardial injury, their specificity for type 1 acute MI is limited (18). Elevated troponins are often found in patients with chronic kidney disease, structural heart disease, or other comorbidities, further complicating interpretation of the results (19–21).

In contrast, 40% of patients in our cohort were classified as excluded, and the majority (80%) were discharged directly from the emergency department. This discharge rate is slightly lower than approximately 60% exclusion rates reported in European cohorts (22, 23), a difference that may reflect underlying population risk profiles or more conservative local clinical practices. In our practice, physicians often admitted patients who met exclusion criteria while other characteristics (such as ECG changes, comorbidity burden, or perceived clinical risk) were concerning. This finding highlights an important principle: Algorithmic classification can guide but cannot replace clinical judgment. The ultimate disposition decision depends not only on biochemical thresholds but also on a more extensive clinical evaluation.

However, the most striking feature of our cohort was the large size of the observation group, which comprised half of all patients and was associated with the longest emergency department length of stay. This reflects the heterogeneity of patients with borderline troponin changes, ambiguous ECG findings, or multiple comorbidities. Unlike the inclusion and exclusion groups, which generally offer clear management pathways, the observation area often requires prolonged monitoring, serial blood draws, and cardiology input before a safe decision can be made. This reality may mitigate the expected efficiency gains from future accelerated diagnostic protocols. Furthermore, it confirms that the management of intermediate-risk patients remains a cautious approach in the early evaluation of suspected ACS.

Our subgroup analysis results highlighted important limitations of the algorithm when applied to higher-risk populations. Nearly one-third of patients with known CAD were included in the confirmed diagnosis group, compared with <5% of those without CAD (13, 24). These patients were also more likely to be diagnosed with MI. This finding is not surprising, as structural heart disease and a history of prior infarction increase the likelihood that troponin elevations reflect acute ischaemia rather than chronic injury. For patients with CAD, the algorithm tended to place a significant proportion of patients in the high-risk zone, reducing its ability to support early discharge. For clinicians, this suggests that the algorithm may have limited utility in those most in need of diagnostic certainty, as preexisting medical conditions can diminish the discriminatory value of troponin changes.

Age also showed a similar effect. Among younger patients under 55 years old, more than half were classified as rule-out, with only a very small number designated for admission. In contrast, among older patients, the majority were clustered in the observation group, with 12% meeting admission criteria. This may be due to the increased troponin concentrations with age, the greater prevalence of comorbidities, and the higher pretest probability of a coronary event (25). This raises the possibility that while the algorithm may offer reassurance for younger, lower-risk patients, its discriminatory power diminishes with age, borderline results become more difficult to interpret, and the observation group size rapidly swells.

In sensitivity analyses, application of the 2020 ESC 0/1 h threshold resulted in a higher proportion of patients classified as confirmed than in the 2015 version, with a concomitant reduction in the size of the observation group. However, in our cohort, this shift did not appear to improve discrimination for myocardial infarction and may have increased the false-positive rate. The specificity observed for the 2015 ESC algorithm in our cohort is higher than the pooled estimate of approximately 91% reported by a meta-analysis (26). This difference likely reflects methodological and population factors, including single-centre adjudication of final diagnoses, fewer patients with chronic troponin elevations, and smaller sample size within the rule-in category. These findings raise the broader question of whether thresholds derived primarily in European validation cohorts are fully applicable to our population. Comorbidities, elevated background troponins, and differences in institutional admission practices may contribute to the observed differences. Therefore, careful consideration and possible recalibration are needed before generalising European thresholds to other healthcare settings.

The 2023 ESC guideline for the management of acute coronary syndromes reaffirmed the 0 h/1 h as the preferred strategy. While the numeric thresholds were not revised from the 2020 version, the update placed stronger emphasis on integrating serial troponin dynamics with ECG interpretation and structured clinical risk assessment and on local calibration of assay performance. This evolution highlights that diagnostic algorithms should not be applied in isolation; rather, their accuracy depends on patient selection, timing of sampling, and contextual clinical judgement. Our findings reinforce this principle, supporting guideline recommendations that population-specific validation and continuous audit are essential when implementing rapid rule-out pathways in diverse healthcare settings.

This study has several limitations. First, the study was conducted at a single centre, and the patient case mix and local admission practices may differ from those in other healthcare systems, limiting the external validity of our findings. Second, admissions and discharges were ultimately determined by individual clinicians, guided by ECG findings, comorbidity burden, and overall clinical judgment, making it difficult to fully separate these influences from the apparent performance of the algorithm. Then, we did not have access to complete medication data, including use of antiplatelet therapy, statins, β-blockers, or other cardioprotective agents. This is important because background therapy influences both baseline troponin levels and clinical outcomes. Finally, as this study used retrospective data, we are only able to access in-hospital data and did not have follow-up results, such as 30-day mortality or major adverse cardiovascular events, which limits the assessment of prognostic safety.

## Conclusion

In this real-world cohort, the ESC 0/1 h hs-cTnT algorithm demonstrated good diagnostic concordance in the rule-in and rule-out zones, but nearly half of patients were classified to the observation group, where uncertainty and prolonged stays persisted. The positive predictive value of the rule-in zone was high but not absolute, reflecting the limited specificity of troponin for type 1 myocardial infarction in a comorbid population. Importantly, reclassification under the 2020 thresholds modestly shifted patients from observation to rule-out, but without eliminating the central challenge of the intermediate group. Together, these findings highlight both the strengths and the limitations of applying European-derived thresholds in our setting, raising the question of whether locally adapted cutoffs may better balance safety and efficiency. Future work should focus on refining risk stratification for observation-zone patients and evaluating whether population-specific thresholds can improve the clinical utility of accelerated diagnostic pathways.

## Data Availability

The original contributions presented in the study are included in the article/Supplementary Material; further inquiries can be directed to the corresponding author.
